# Supplementary figures and images for: The utility of urinary biomarker panel in predicting renal pathology and treatment response in Chinese lupus nephritis patients
Source: PLoS One. 2020 Oct 27;15(10):e0240942. doi: 10.1371/journal.pone.0240942 (PMC7591050; doi:10.1371/journal.pone.0240942)

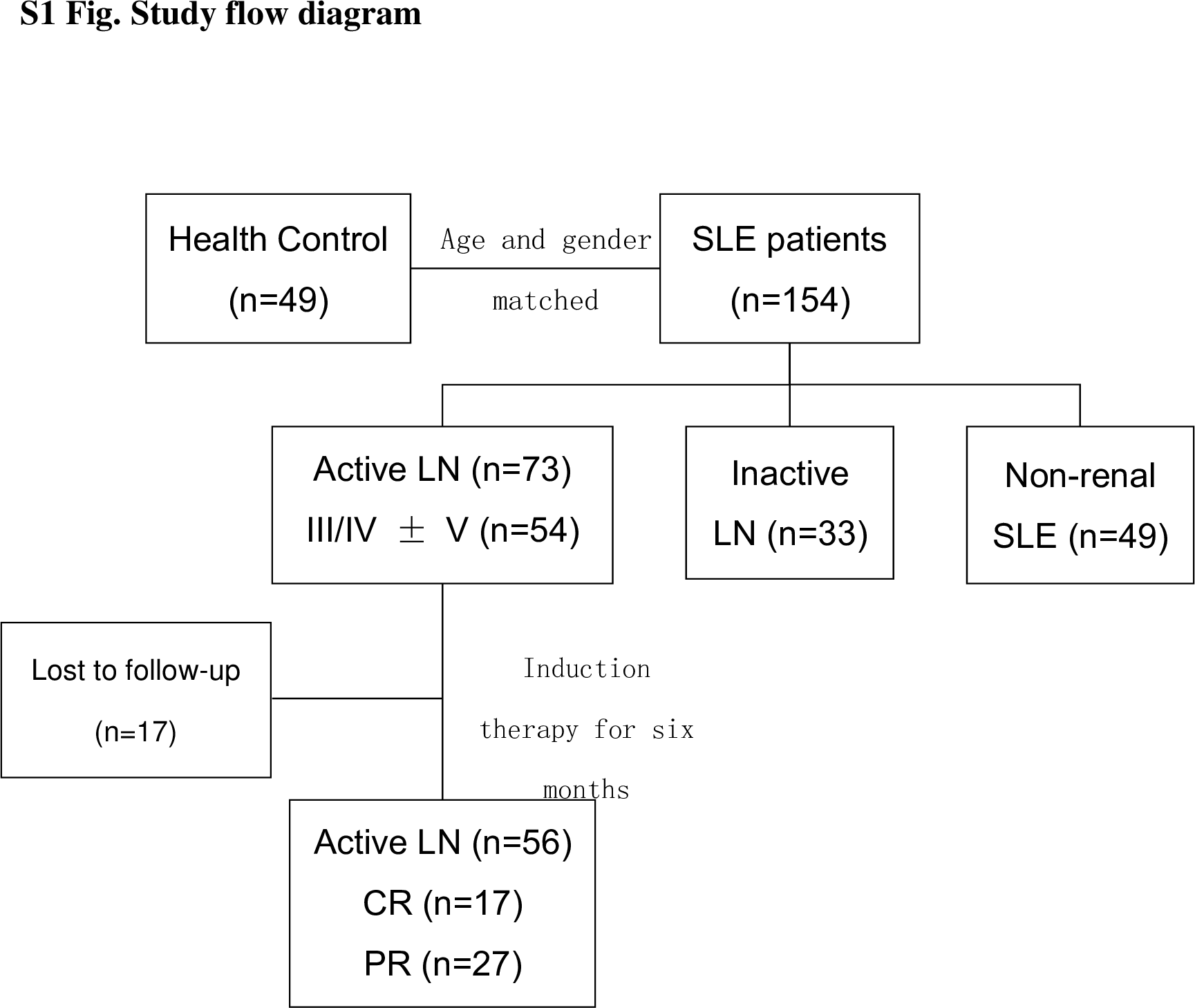

Supplement: S1 Fig — (TIF) [file pone.0240942.s001.tif]
