# Supplementary material for: The utility of urinary biomarker panel in predicting renal pathology and treatment response in Chinese lupus nephritis patients
Source: PLoS One. 2020 Oct 27;15(10):e0240942. doi: 10.1371/journal.pone.0240942 (PMC7591050; doi:10.1371/journal.pone.0240942)
Supplement: S1 Table — (DOCX) [file pone.0240942.s002.docx]

**S1 Table. Baseline characteristics of patients with different renal responses**

|  | **CR**  **(n = 17)** | **PR**  **(n = 27)** | **NP**  **(n = 12)** |  |
| --- | --- | --- | --- | --- |
| Sex (F/M) n | 15/1 | 25/2 | 12/1 |  |
| Age (years) Mean±SD | 36.24±13.85 | 39.07±15.55 | 31.67±10.10 |  |
| **Clinical Manifestations** | | | |  |
| Fever n(%) | 2(11.76) | 1(3.70) | 1(8.33) |  |
| Rash n(%) | 7(41.18) | 3(11.11) | 2(16.67) |  |
| Vasculitis n(%) | 0(0) | 0(0) | 1(8.33) |  |
| Ulcer n(%) | 5(29.41) | 3(11.11) | 2(16.67) |  |
| Serositis n(%) | 2(11.76) | 2(7.41) | 0(0) |  |
| Arthritis n(%) | 6(35.29) | 6(22.22) | 1(8.33) |  |
| NPSLE n(%) | 0(0) | 0(0) | 0(0) |  |
| Nephritis n(%) | 17(100) | 27(100) | 12(100) |  |
| PAH n(%) | 0(0) | 0(0) | 0(0) |  |
| Hematologic n(%) | 2(11.76) | 3(11.11) | 2(16.67) |  |
| **Laboratory test** | | | |  |
| WBC（10^9/L) Median(IQR) | 7.62(6.08-9.50) | 7.50(4.22-10.47) | 5.60(3.74-7.38) |  |
| Hb (g/L) Mean±SD | 105.20±20.46 | 110.20±22.56 | 104.10±23.35 |  |
| Plt (10^9/L) Median (IQR) | 221 (165-280) | 195(151-258) | 182(128-223) |  |
| ESR (mm/h) Median (IQR) | 28(9-53) | 23(13-49) | 32(12-42) |  |
| CRP (mg/L) Median (IQR) | 3.13(3.00-3.61) | 3.16(1.30-7.68) | 3.11(3.02-3.16) |  |
| MDRD-GFR (mL/(min*1.73m2)) Mean±SD | 94.23±40.23 | 101.8±50.03 | 98.59±37.32 |  |
| Serum Creatinine (μmol/L) Median (IQR) | 70(51-102) | 59(53-89) | 66(54-88） |  |
| 24h urine protein (g/24h) Median (IQR) | 1.75 (0.92-5.39) | 2.56(1.33-4.16)- | 2.69(1.44-5.94) |  |
| Complement C3 (g/L) Mean±SD | 0.67±0.26 | 0.61±0.28 | 0.56±0.24 |  |
| Complement C4 (g/L) Median (IQR) | 0.13(0.06-0.20) | 0.08(0.04-0.13) | 0.12(0.07-0.19) |  |
| dsDNA (IU/ml) Median (IQR) | 34.29(11.47-100.00) | 70.23(29.00-100.0) | 12(6.46-59.42) |  |
| **Autoantibodies, n of positive subjects (%)** | | | |  |
| Anti-Sm | 2(11.76) | 3(11.11) | 1(8.33) |  |
| Anti-SSA/Ro | 11(64.71) | 12(44.44) | 6(50.00) |  |
| Anti-RNP | 4(23.53) | 13(48.15) | 1(8.33) |  |
| Anti-SSB | 3(17.65) | 3(11.11) | 0(0) |  |
| Anti- Nucleosome | 4(23.53) | 15(55.55) | 2(16.67) |  |
| Anti- Ribosomal -P | 2(11.76) | 6(22.22) | 1(8.33) |  |
| Anti- Histone | 2(11.76) | 5(18.52) | 1(8.33) |  |
| APL | 0(0) | 1(3.70) | 0(0) |  |
| SLEDAI, Median (IQR) | 10(8-14) | 10(8-12) | 9(6-12) |  |
| RSLEDAI, Median (IQR) | 4(4-4) | 8(4-8) | 4(4-8) |  |
| **Medications** | | | | |
| Pred (mg) Median (IQR) | 22(11-30) | 43(30-60) | 30(23-45) |  |
| Methotrexate n (%) | 0(0) | 0(0) | 0(0) |  |
| Azathioprine n (%) | 0(0) | 0(0) | 0(0) |  |
| CsA n (%) | 2(11.76) | 3(11.11) | 1(8.33) |  |
| Tacrolimus n (%) | 2(11.76) | 3(11.11) | 0(0) |  |
| Leflunomide n (%) | 0(0) | 1(3.70) | 0(0) |  |
| MMF n (%) | 2(11.76) | 3(11.11) | 4(33.33) |  |
| CYC n (%) | 10(58.82) | 17(62.96) | 5(41.67) |  |
| Iguratimod n (%) | 1(5.88) | 0(0) | 1(8.33) |  |
| Thalidomide n (%) | 0(0) | 0(0) | 1(8.33) |  |
| **Histological type** | | | |  |
| Proliferative LN n (%) | 11(64.71) | 23(85.19) | 11(91.67) |  |
| Class III, IV, III+V, IV+V n | 3,5,1,2 | 1,13,6,3 | 2,3,1,5 |  |
| AI Median (IQR) | 4(2-9) | 7(3-9) | 8(5-10) |  |
| CI Median (IQR) | 3(2-6) | 4(3-6) | 6(2-8) |  |
| Membranouse LN n (%) |  |  |  |  |
| Class V n | 6(35.29) | 4(14.81) | 1(8.33) |  |
| AI Median (IQR) | 1(1-1) | 1(0-1) | 1 |  |
| CI Median (IQR) | 3(2-3) | 4(3-5) | 3 |  |

CR: comelete response; PR: partial response; NR: Non-response; SD, standard deviation; F, female; M, male; NPSLE, Neuropsychiatric systemic lupus erythematosus; PAH, pulmonary arterial hypertension; WBC, white blood cell; Hb, hemoglobin; Plt, platelet; ESR, erythrocyte sedimentation rate; CRP, C-reactive protein; MDRD, modification of diet in renal disease Study; GFR, glomerular filtration rate; dsDNA, anti-double-stranded DNA antibody; anti-Sm, anti-Smith; Anti-SSA/Ro, Anti-Sjögren’s-syndrome-related antigen A/Ro; anti-RNP anti-ribonucleoprotein; Anti-SSB, anti-Sj gren syndrome B; APL, anti-phorpholipid; SLEDAI, SLE disease activity index; RSLEDAI, renal SLEDAI; AI, Activity Index; CI, Chronicity Index; Pred: prednisone; CsA: cyclosporin A; MMF: mycophenolate mofetil; CYC: cyclophosphamide.
